# The EMMY longitudinal, cohort study: real-world data to describe multiple myeloma management and outcomes as more therapeutic options emerge

**DOI:** 10.46989/001c.121371

**Published:** 2024-07-23

**Authors:** Olivier Decaux, Ronan Garlantézec, Karim Belhadj-Merzoug, Margaret Macro, Laurent Frenzel, Aurore Perrot, Philippe Moreau, Bruno Royer, Denis Caillot, Xavier Leleu, Mohamad Mohty, Lionel Karlin, Pierre Feugier, Sophie Rigaudeau, Jean Fontan, Cécile Sonntag, Laure Vincent, Thomas Chalopin, Herve Avet Loiseau, Zakaria Maarouf, Louni Chanaz, Nathalie Texier, Cyrille Hulin

**Affiliations:** 1 Service d’hématologie clinique Centre Hospitalier Universitaire de Rennes; 2 Santé publique et épidémiologie Centre Hospitalier Universitaire de Rennes; 3 Unité Fonctionnelle Hémopathies Lymphoïdes Centre Hospitalier Universitaire Henri-Mondor; 4 Service d’hématologie clinique Centre Hospitalier Universitaire de Caen; 5 Service d’hématologie adulte Hôpital Necker-Enfants Malades; 6 Hématologie Centre Hospitalier Universitaire de Toulouse; 7 Hématologie clinique CHRU - Hôtel Dieu (Nantes); 8 Immuno-Hématologie Hôpital Saint-Louis (Paris); 9 Hématologie Clinique Centre Hospitalier Universitaire Dijon Bourgogne; 10 Hématologie Centre Hospitalier Universitaire de Poitiers; 11 Service d’Hématologie et Thérapie cellulaire Hôpital Saint-Antoine; 12 Hématologie clinique Hospices Civils de Lyon; 13 Service d’hématologie CHU de Nancy - Hôpitaux de Brabois; 14 Service d’Hématologie CHV André Mignot; 15 Service Hématologie CHRU Jean Minjoz (Besançon); 16 Département d’Hématologie et Oncologie Hôpitaux Universitaires de Strasbourg; 17 Département d’Hématologie et Oncologie Hôpital de Hautepierre et Hôpital Civil; 18 Département d’hématologie clinique CHU Montpellier; 19 Hématologie thérapie cellulaire CHRU de Tours; 20 IUCT Oncopole Toulouse; 21 CHU Toulouse; 22 Intergroupe Francophone du Myélome; 23 Epidémiologie Kappa Santé; 24 Hématologie Hôpital Haut-Lévêque (Pessac)

**Keywords:** Multiple myeloma, real-world data, therapeutic management

## Abstract

The therapeutic management of patients with multiple myeloma (MM) is complex. Despite substantial advances, MM remains incurable, and management involves cycles of treatment response, disease relapse, and further therapy. Currently, evidence to support the therapeutic decision is limited. Thus, the EMMY longitudinal, real-world study was designed to annually assess therapeutic management of MM in France to provide evidence to support physicians. During an annual prespecified 3-month recruitment period, eligible patients will be identified from their medical records. Adults aged ≥18 years diagnosed with symptomatic MM and requiring systemic treatment will be eligible. The primary objective, the evolution of MM therapeutic management, will be described, as well as the impact on the following outcomes: time-to-next treatment (TTNT), progression-free survival (PFS), and overall survival (OS). The study plans to recruit 5000 patients over 6 years: 700 to 900 patients annually. EMMY is a unique opportunity to collect real-world data to describe the evolving MM therapeutic landscape and record outcomes in France. These data will provide annual snapshots of various aspects of MM management. This knowledge will provide physicians with real-life, evidence-based data for therapeutic decision-making and ultimately improve treatment for MM patients.

## Introduction

Multiple myeloma (MM) is a hematological B-cell malignancy characterized by abnormal proliferation of clonal bone marrow plasma cells, a type of terminally differentiated B-cell.^1–3^ This causes overproduction of monoclonal immunoglobulins, including complete IgG, IgA, IgM, or only light chains, which damage end-organs and are associated with immune dysfunction.^1–3^ End-organ damage often includes hypercalcemia, renal failure, anemia, and/or bone lesions, the so-called CRAB criteria.^1,4^

MM is the second most frequently reported hematological malignancy. In 2020, 176,404 new cases of MM were reported and caused 117,077 deaths.^5,6^

Despite the substantial therapeutic advances made over the last two decades, MM remains incurable.^2^ However, recent advances have extended progression-free survival (PFS) and overall survival (OS).^7^ Today, MM management involves reiterative stages of response to treatment, inevitable disease relapse, and initiation of subsequent lines of therapy.^2,3^

Numerous therapies for treating MM are available, including proteasome inhibitors, alkylating agents, immunomodulatory imide drugs, and monoclonal antibodies.^3,8–12^ Moreover, therapies targeting B-cell maturation antigens have been developed, including antibody-drug conjugates, bispecific antibodies (BiAbs), bispecific T-cell engagers, and chimeric antigen receptor T (CAR-T) cells.^13,14^ More recently, therapeutic agents (including BiAbs and CAR T cells) targeting G protein-coupled receptor class C group 5 member D have shown efficacy.^15,16^ In addition, autologous stem-cell transplantation (ASCT) is the backbone of MM management. In patients eligible for ASCT, induction therapy with bortezomib and dexamethasone combined with daratumumab-thalidomide is recommended. This is followed by melphalan then ASCT. The recommended frontline therapy for ASCT-eligible patients is completed by maintenance with lenalidomide. In patients not eligible for ASCT, various frontline combination therapies are available, but currently, the combination of daratumumab, lenalidomide, and dexamethasone is the most effective regimen.^17^ At inevitable relapse, the choice of therapy depends on several factors, including age, comorbidities, MM symptoms, and prior treatments, as well as the availability of therapies.^1^ The emergence of new treatments will further complexify the therapeutic decision.

## Materials and Methods

### Study design

EMMY is a multicenter, non-interventional, historical, longitudinal cohort study. Real-life data will be collected in centers specialized in MM management and members of the French myeloma intergroup, “Intergroupe Francophone du Myélome (IFM)”. The study was funded by the IFM with support from a consortium of pharmaceutical companies. The EMMY Scientific Committee members, independently of contributors, defined and conducted the writing of this EMMY publication. Most patients with MM are managed in IFM centers. Seventy-three centers have been selected: 34 general hospitals, 26 university hospitals, 11 private hospitals/clinics, 1 military hospital, and 1 comprehensive cancer center (Supplemental Table 1). If required, additional centers can be included. Overall, the average potential patient population in these centers is 46 patients per day per center.

**Figure 1.**
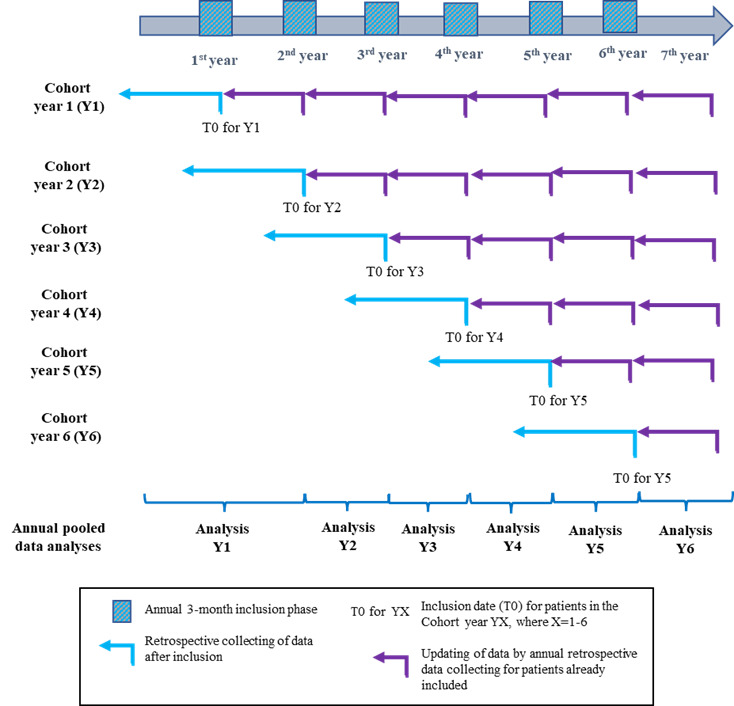
Overall study design

Patients are recruited over 6 years (2017-2023) during annual, pre-defined, 3-month periods (see Figure 1), and data are updated annually.

### Study population

Patients will be identified from their medical records at the centers. Adults aged ≥18 years, diagnosed with symptomatic MM and requiring systemic treatment (irrespective of the treatment line) will be eligible. Patients with non-secretory, solitary plasmacytoma, or plasma cell leukemia will be excluded.

### Study objectives

The primary objective of the EMMY study is to describe the therapeutic management of patients with MM in French hospitals using real-life data.

Secondary objectives aim to assess PFS, TTNT, OS and treatment response rates, as well as to assess therapy discontinuations and the evolution of MM practices. The study will also assess the impact of MM on healthcare resources and on patients’ professional activity, as well as the evolution of healthcare resource utilization, using the study data and data retrieved from the French healthcare database, the “Système National des Donnes de Santé” (SNDS).

### Study endpoints

The study aims to describe the evolution of MM therapeutic management and its impact on the following outcomes: time-to-next treatment (TTNT), PFS, and OS.

Secondary endpoints will be therapy administered according to age, gender, body mass index, Eastern Cooperative Oncology Group (ECOG) performance status, co-morbidities, medical history, clinical and biochemical parameters, as well as MM symptoms and characteristics. MM treatment modalities will be described according to the time interval between diagnosis and treatment initiation, prior treatments and refractoriness to these, reason for initiation, treatment dosage at initiation and during follow up.

### Data collection

Once patients have been informed and do not oppose to participating, they will be enrolled in the study and data will be retrieved from their medical files. No additional data nor study specific visits are planned. Each year, selected data on treatment response and medical history will be retrospectively updated (**Figure 1**) The data to be collected and the collection process are shown in **Table 1**.

**Table 1.** Patient data collected during the EMMY study

***Data collected***		***Newly recruited patients***	***Annual update of data***
Sociodemographic	Age, gender, height, weight, and ECOG PS	X	X
Multiple myeloma diagnosis	Date of diagnosis	X	
	Type of myeloma (at diagnosis)	X	
	Immunoglobulin type	X	
	ISS stage (at diagnosis)	X	
Multiple myeloma diagnostic criteria	CRAB criteria	X	
	Malignancy biomarkers	X	
Multiple myeloma treatment(s) and response(s)	Radiotherapy	X	X
	Corticosteroid therapy	X	X
	Autograft(s) and allograft(s)	X	X
	Multiple myeloma treatment(s)	X	X
	Response(s) to treatment(s)	X	X
	Date of discontinuation for each line of treatment with reason	X	X
Cytogenetics	Del13q, t(4;16), t(4;14), Del17p, and others	X	
Medical history and comorbidities	History of myeloma (familial immunoglobulin G, MGUS, SMM etc.)	X	
	History of cancer (including leukaemia, solid cancers, lymphoma etc.)	X	
	Other relevant medical history	X	X
	Concomitant diseases (including diabetes, HIV, and cardiovascular, renal, liver, and pulmonary diseases)	X	X
Biological data	Full blood count	X	X
	Biochemistry	X	X
	Immunoglobulin assays	X	X
	Immunofixation	X	X
Imaging data	Type of imaging (X-rays, scans, MRIs, PET-CT)	X	X
	Lesions identified (including plasmacytomas)	X	X
Study withdrawal	Date and reason for study withdrawal (termination of data collection for the given patient)		X

CRAB: hypercalcaemia, renal failure, anaemia, and/or bone lesions, ECOG PS: Eastern Cooperative Oncology Group Performance Status, HIV: human immunodeficiency virus, ISS: international staging system, MGUS: monoclonal gammopathy of undetermined significance, MM: multiple myeloma, SMM: smoldering multiple myeloma, MRI: magnetic resonance imaging, PET-CT: positron emission tomography–computed tomography.

In addition to patient information, data on the investigators and the healthcare institutions will be collected to assess study representativeness. Investigator data include their specialty (hematologist, oncologist, rheumatologist, internal medicine physician, etc.) and sociodemographic values (gender, age, medical experience). The institution data include the type of healthcare institution and the number of MM patients treated.

Furthermore, data will be retrieved from the SNDS and associated with the ancillary study’s clinical data. The SNDS and clinical data will be linked by probabilistic chaining using indirect nominative variables for each patient. The data collected from the SNDS will include details on hospitalizations, visits to the emergency department without hospitalizations, medical consultations, medical procedures and examinations (such as imagery and laboratory tests), paramedical procedures, treatments, and medical transport.

### Data management

Patient data will be collected at each center using an electronic case report form (e-CRF) and centralized in the study database. Critical data will be mandatory in the e-CRF. Data coherence and consistency will be verified throughout the study. Data quality control will be performed at least annually by the scientific committee during a review meeting before the database locks. The steering, scientific, and operational committees will oversee the study.

### Sample size and statistical analysis

Overall, we plan to recruit approximately 5,000 patients with symptomatic MM, ranging from 700 to 900 patients annually.

The data will be analyzed annually to obtain a “snapshot” of MM management during the previous year. Data will be summarized using descriptive statistics (numbers with percentages, means with standard deviations, or medians with 95% CI) and presented graphically to highlight trends. The number of missing data points will be indicated. Time-to-event analyses (OS and PFS) will be performed using the Kaplan-Meier method, which will be reported as medians with 95% CIs and presented as plots. The analysis’s significance level is set at 5% unless otherwise specified.

PFS is the interval between treatment initiation and disease progression or death, whichever occurs first. TTNT is the interval between the treatment initiation of a given treatment line and the subsequent treatment line or death. OS is the time interval between treatment initiation and death of any cause. Response rates will be the proportion of patients in each response category at the time point of interest. The best response for each line of treatment will be retained for the analyses. PFS and response will be assessed using the International Myeloma Working Group definitions.^18^ Treatment discontinuation will include the time interval from treatment initiation to discontinuation and the reason(s) for discontinuation. The outcomes will be described according to the line of treatment, patient profiles, and other criteria of interest (defined by the scientific committee). Lastly, the evolution of MM management practices will be assessed and described, including therapy choice, use of grafts, hospitalizations, follow-up, and examinations performed.

Data management and statistical analysis will be performed using SAS (version 9.1, SAS Institute, North Carolina, USA).

The study is sponsored by the IFM group and financed and overseen by a consortium of partners that represent key role players in MM management in France.

## Discussion

Over the last few decades, therapeutic advances have improved outcomes for patients with MM. However, the extensive arsenal of new treatments approved and in clinical development has complexified therapeutic decision-making for physicians. Indeed, physicians lack data to make and support these decisions.

EMMY is a unique opportunity to collect real-world data to describe and evaluate France’s evolving MM therapeutic management. The study will collect real-world data annually from a representative sample of MM patients.

The EMMY study design, with an annual 3-month recruitment period over 6 years, will collect patient data, MM diagnosis and disease evolution, therapeutic management, and survival outcomes. The analyses will provide annual “snapshots” of MM prevalence and therapeutic management. This will detect treatment strategies that depend on age, disease severity, prior treatments, and the availability of new treatments. In addition, the clinical data will be associated with SNDS data to access healthcare resource utilization and obtain a more complete overview of MM management.

Two international observational studies have assessed aspects of MM management. The CoMMpass study (NCT01454297) predominantly focused on identifying the clinical characteristics and molecular profiles of patients with MM, while the INSIGHT MM cohort (NCT02761187) collected data on various aspects of MM, as in our study. However, the latter study was completed in 2021,^18^ and the EMMY study will provide more recent data concerning the therapeutic management of patients with MM.

As with all real-world studies, this study is limited by data quality. The data quality in the medical files will depend on its completeness and accuracy.

Overall, the EMMY study is a unique and important collaboration between various key players in MM management in France. Our common objective is to provide physicians with evidence-based data for therapeutic decisions and, ultimately, to improve treatment for MM patients.

### Authors’ Contribution

Conceptualization: Olivier Decaux (Equal), Ronan Garlantézec (Equal), Karim Belhadj-Merzoug (Equal), Margaret Macro (Equal), Laurent Frenzel (Equal), Aurore Perrot (Equal), Philippe Moreau (Equal), Bruno Royer (Equal), Laure Vincent (Equal), Thomas Chalopin (Equal), Herve Avet Loiseau (Equal), Cyrille Hulin (Equal). Writing – review & editing: Olivier Decaux (Equal), Ronan Garlantézec (Equal), Karim Belhadj-Merzoug, Margaret Macro (Equal), Laurent Frenzel (Equal), Aurore Perrot (Equal), Philippe Moreau (Equal), Bruno Royer (Equal), Denis Caillot (Equal), Xavier Leleu (Equal), Mohamad Mohty (Equal), Lionel Karlin (Equal), Pierre Feugier (Equal), Sophie Rigaudeau (Equal), Jean Fontan (Equal), Cécile Sonntag (Equal), Laure Vincent (Equal), Thomas Chalopin (Equal), Herve Avet Loiseau (Equal), Zakaria Maarouf (Equal), Louni Chanaz (Equal), Nathalie Texier (Equal), Cyrille Hulin (Equal). Methodology: Ronan Garlantézec (Lead).

### Competing of Interest

Olivier Decaux declares receiving honoraria from Bristol Myers Squib (BMS), Gilead, GlaxoSmithKline (GSK), Janssen, Roche, Sanofi, and Takeda. Karim Belhadj-Merzoug received honoraria, research funding, and traveling expenses from Amgen, BMS, Janssen, Pfizer, Sanofi, and Takeda. Margaret Macro received honoraria and research funding from Amgen, BMS, GSK, Janssen, Sanofi, and Takeda.  Laurent Frenzel received consulting fees and research funding from BioMarin, CSL Behring, Pfizer, Sobi, and Roche. Aurore Perrot received honoraria from AbbVie, Adaptive, Amgen, BMS, Janssen, Pfizer, Sanofi, and Takeda. Philippe Moreau received honoraria and payment for advisory boards from AbbVie, Amgen, BMS-Celgene, Janssen, Pfizer, Sanofi, and Takeda. Xavier Leleu received consultancy fees from AbbVie, Amgen, BMS, Gilead, GSK, Harpoon Therapeutic, iTeos therapeutics, Janssen, Merck, Novartis, Oncopeptides, Pfizer, Regeneron, Roche, Sanofi, and Takeda. Mohamad Mohty received honoraria, consultancy fees, and research funding from Amgen, BMS, Gilead, GSK, Janssen, Jazz Pharmaceuticals, Novartis, Pfizer, Sanofi, Stemline Therapeutics, and Takeda. Lionel Karlin received honoraria, payment for advisory boards, as well as logistical and financial assistance for attending conferences from AbbVie, Amgen, BMS-Celgene, GSK, Janssen, Pfizer, Sanofi, Stemline Therapeutics, and Takeda. Cécile Sonntag is a member of the board of directors or on the advisory committee of BMS, Janssen, Sanofi, and Takeda. Laurent Vincent declares being a board member for BMS and Takeda. He also declares that he is receiving honoraria from Janssen and Pfizer. Cyrille Hulin received honoraria from AbbVie, Amgen, BMS, Janssen, Pfizer, and Sanofi. The other authors have no competing interests to declare.

### Ethical Conduct Approval

The EMMY study is classified as research not involving human subjects, as defined in article L. 1121-1 of the French Public Health Code. This retrospective and prospective personal data study is being conducted following the Declaration of Helsinki and has been submitted to the French Health Data (N° F20220518161845) https://www.health-data-hub.fr/projets/emmy-epidemiologie-de-laprise-en-charge-therapeutique-du-myelome-multiple-en-france No ethical approval for this study was required. All patients were informed about the processing of their data before enrolment in the study.

### Informed Consent Statement

All authors and institutions have confirmed this manuscript for publication.

### Data Availability Statement

Not applicable.
